# Cold chain and virus‐free oral polio booster vaccine made in lettuce chloroplasts confers protection against all three poliovirus serotypes

**DOI:** 10.1111/pbi.13060

**Published:** 2019-01-11

**Authors:** Henry Daniell, Vineeta Rai, Yuhong Xiao

**Affiliations:** ^1^ Department of Biochemistry School of Dental Medicine University of Pennsylvania Philadelphia PA USA

**Keywords:** human infectious diseases, lettuce chloroplast transformation, bioencapsulation, oral delivery, Polio viral protein 1, mucosal immunity

## Abstract

To prevent vaccine‐associated paralytic poliomyelitis, WHO recommended withdrawal of Oral Polio Vaccine (Serotype‐2) and a single dose of Inactivated Poliovirus Vaccine (IPV). IPV however is expensive, requires cold chain, injections and offers limited intestinal mucosal immunity, essential to prevent polio reinfection in countries with open sewer system. To date, there is no virus‐free and cold chain‐free polio vaccine capable of inducing robust mucosal immunity. We report here a novel low‐cost, cold chain/poliovirus‐free, booster vaccine using poliovirus capsid protein (VP1, conserved in all serotypes) fused with cholera non‐toxic B subunit (CTB) expressed in lettuce chloroplasts. PCR using unique primer sets confirmed site‐specific integration of CTB‐VP1 transgene cassettes. Absence of the native chloroplast genome in Southern blots confirmed homoplasmy. Codon optimization of the VP1 coding sequence enhanced its expression 9–15‐fold in chloroplasts. GM1‐ganglioside receptor‐binding ELISA confirmed pentamer assembly of CTB‐VP1 fusion protein, fulfilling a key requirement for oral antigen delivery through gut epithelium. Transmission Electron Microscope images and hydrodynamic radius analysis confirmed VP1‐VLPs of 22.3 nm size. Mice primed with IPV and boosted three times with lyophilized plant cells expressing CTB‐VP1co, formulated with plant‐derived oral adjuvants, enhanced VP1‐specific IgG1, VP1‐IgA titres and neutralization (80%–100% seropositivity of Sabin‐1, 2, 3). In contrast, IPV single dose resulted in <50% VP1‐IgG1 and negligible VP1‐IgA titres, poor neutralization and seropositivity (<20%, <40% Sabin 1,2). Mice orally boosted with CTB‐VP1co, without IPV priming, failed to produce any protective neutralizing antibody. Because global population is receiving IPV single dose, booster vaccine free of poliovirus or cold chain offers a timely low‐cost solution to eradicate polio.

## Introduction

Two types of polio vaccine are currently used around the globe. The live attenuated Oral Polio Vaccine (OPV) developed by Sabin is easy to administer, less expensive and offers gastrointestinal mucosal immunity, which is important in countries with an open sewer system that causes reinfection. However, genetic instability results in neuro‐virulence by reversion of mutations in the attenuated vaccine strains, causing vaccine‐associated paralytic poliomyelitis in the vaccine recipients or their contacts. Further complicating this situation is the recombination that might occur during replication of polio and non‐polio human enteroviruses, resulting in circulating vaccine‐derived polioviruses (VDPVs – Minor, 2009). Another vaccine is the formaldehyde inactivated poliovirus (IPV) developed by Salk which is more expensive, does not offer gastrointestinal mucosal immunity and is predominantly used in countries with closed sewer systems, with minimal reinfection. IPV confers effective humoural immunity but does not prevent replication of WT poliovirus, resulting in poliovirus transmission within a population. Detection of wild poliovirus (WPV) throughout Israel sewer system after prolonged use of IPV, offers proof for such transmission (Yaari *et al*., 2016). Thus, poliovirus is reintroduced into countries where this disease has been eradicated by travellers or VDPVs but Israel is one among the few countries that evaluated contamination of such wild poliovirus in the sewer system. Production of both OPV and IPV vaccines requires culturing infectious polioviruses and therefore requires cold chain for storage, transportation and are not free of poliovirus.

Continued use of serotype 2 in oral polio vaccine (OPV) led to 683 cases of polio caused by circulation of vaccine‐derived poliovirus type 2 (cVDPV2) between 2000 and 2014 (McCarthy *et al*., 2017). Therefore, WHO Global Polio Eradication Initiative recommended withdrawal of the type 2 component (OPV2) in April 2016 and replacing bivalent OPV (bOPV) in all 155 countries and territories that had used OPV in 2015 (Holmes *et al*., 2017; Ramirez Gonzalez *et al*., 2017). The Strategic Advisory Group of Experts (SAGE) proposed that during the transition, a single dose of inactivated poliovirus vaccine (IPV) may be adequate to prime global population due to the high cost and inadequate supply of IPV. However, the proposed phasing out of OPV to IPV warrants further research (Byberg and Fisker, 2016). Recent findings suggest that priming mice subcutaneously with single or even double dose of IPV results in decreasing IgG1 titres to background levels, 1 year after immunization (Lund *et al*., 2015; Xiao and Daniell, 2017). When compared to OPV, IPV does not induce adequate gastrointestinal mucosal immunity (Chan *et al*., 2016; Hird and Grassly, 2012). Furthermore, prolonged and exclusive use of IPV leads to silent polio outbreaks (Yaari *et al*., 2016). This is a major setback for eradication of both the wild‐type‐ and vaccine‐derived polio viruses. Therefore, several innovative strategies are discussed in the Global Polio Eradication Initiative (GPEI) plan 2013–2018. Booster vaccines should be continued until long‐term antibody persistence has been demonstrated beyond age two (Rennels, 2009).

One approach could be the use of virus‐like particles (VLPs) as oral boosters following IPV priming to maintain sustained immunoglobulin titres. VLPs are self‐assembled structures that mimic the native viral organization and morphology, with the native antigenic conformation, but lack the viral genome. The VLPs are therefore safer than current polio vaccines that contain live or inactivated polio virus serotypes because they cannot replicate, mutate or recombine but can elicit a strong immune response (Bachmann and Jennings, 2010; Chen and Lai, 2013; Grgacic and Anderson, 2006; Kushnir *et al*., 2012; Marsian and Lomonossoff, 2016; Roldao *et al*., 2010). The major setback in the commercialization of VLPs, however, is their high cost of production, purification, stability, injections and cold chain for storage and transportation. There is at least one report of polio VLP production of PV3 in tobacco through agro‐infiltration; the processed P1 assembled as VLPs and retained the native antigenic conformation but lacked infectious genomic material. Injection of mice with purified VLPs protected them from poliovirus challenge (Marsian *et al*., 2017). Although this approach is virus‐free, lack of mucosal immunity or need for purification, injection and cold chain does not address problems associated with other polio vaccines.

Oral delivery of vaccine antigens expressed in lettuce chloroplasts offers another approach that eliminates culturing viruses, cold chain, prohibitively expensive purification and injections. Biopharmaceutical and vaccines expressed in chloroplasts are stable at ambient temperature for many years with maintenance of protein folding and therapeutic efficacy (Chan *et al*., 2016; Daniell *et al*., 2016; Herzog *et al*., 2017; Su *et al*., 2015). Bioencapsulation of antigens within plant cell wall protects them from degradation from stomach acids/enzymes upon oral delivery; commensal bacterial release enzymes to digest plant cell wall and antigens are then released in the gut lumen (Kwon and Daniell, 2015). Fusion of antigens with GM1 ganglioside receptor‐binding protein CTB (cholera non‐toxic subunit B) facilitates efficient delivery of vaccine antigens to the immune system (Chen *et al*., 2015; Gupta *et al*., 2015; Kumar and Daniell, 2004; Kwon and Daniell, 2016; Xiao *et al*., 2016). GM1 is broadly distributed in a variety of cell types including epithelial cells of the gut and antigen‐presenting cells, macrophages, dendritic cells, and B cells allowing the fusion antigens to efficiently cross the mucosal barrier via the GM1‐ganglioside receptor and provides optimal access to the immune system (Baldauf *et al*., 2015; Stratmann, 2015). This delivery system was first developed in tobacco chloroplasts but in the past several years lettuce system has been advanced to express several vaccine antigens including cholera, malaria (Davoodi‐Semiromi *et al*., 2010), dengue (Kanagaraj *et al*., 2011), plague (Arlen *et al*., 2007), tuberculosis (Lakshmi *et al*., 2013) and anthrax (Koya *et al*., 2005; Ruhlman *et al*., 2010). These studies demonstrated generation of high‐level antibody titres, conferring mucosal as well as systemic immunity, and protection against pathogen or toxin challenges. However, it is important for future studies to focus on elimination of the selectable marker antibiotic resistance gene because they are present in thousands of copies in each transplastomic cell, increase metabolic load and prevent reuse of the same selectable marker gene in subsequent transformation for additional genes or traits.

Our laboratory reported expression of CTB‐VP1 in tobacco chloroplasts, enhancing expression through codon optimization (Kwon *et al*., 2016). Subsequently using mass spectrometry‐based parallel reaction monitoring (PRM), we were able to accurately quantify *in planta* antigen concentration (Kwon *et al*., 2016). Oral delivery of lyophilized tobacco cells expressing CTB‐VP1fusion protein induced strong mucosal and systemic immunity, with high antigen‐specific titres of both IgG1 and IgA. Poliovirus neutralization studies performed at Center for Disease Control (CDC) laboratory using sera from immunized animals confirmed protection against all three poliovirus serotypes, with high‐level neutralization titres and seropositivity (Chan *et al*., 2016). However, several major limitations in these studies include use of non‐edible plant system (tobacco) and fifteen boosters during a long‐term (400 days) study (Xiao and Daniell, 2017). In this study, we have expressed CTB‐VP1 in lettuce chloroplasts and reduced the number of boosters to three doses by using oral plant‐based adjuvants (saponin, squalene) and antimicrobial peptides (LL37 and PG1) to enhance immune modulation. For long‐term evaluation, an additional oral booster was given after 300 days. Maintenance of IgG1 and IgA titres and protection against poliovirus challenged with all three serotypes were investigated. Outcome of these investigations augur well for an affordable plant booster vaccine free of poliovirus or cold chain to eradicate polio around the globe.

## Results

### Characterization of CTB‐VP1 transplastomic lettuce lines

**Figure 1:**
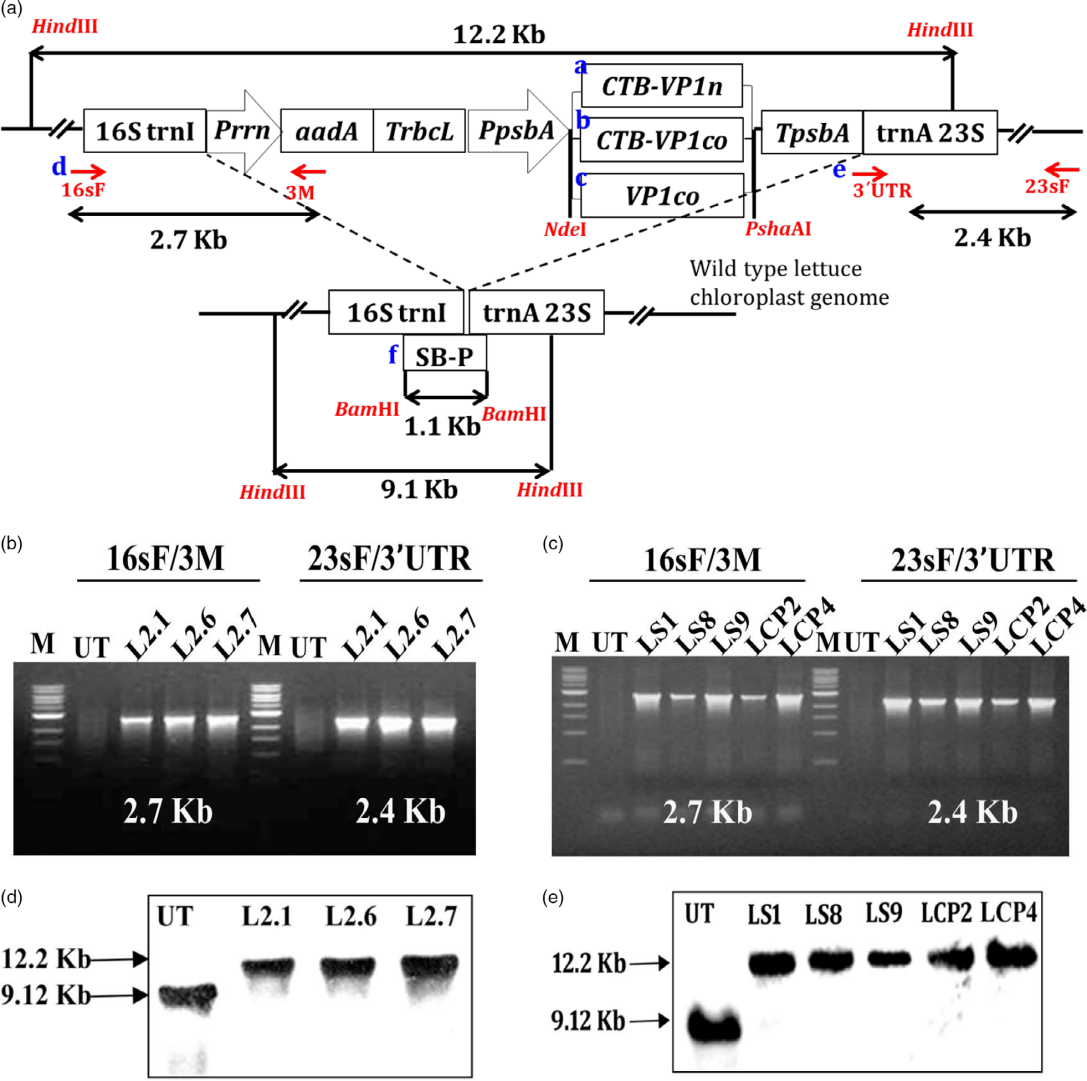
Creation and characterization of transplastomic lettuce plants expressing native and codon‐optimized CTB‐VP1 (a) Lettuce chloroplast transformation vectors containing native (n) and codon‐optimized (co) CTB‐VP1, and VP1co expression cassettes (denoted as a, b, c). Prrn, rRNA operon promoter; aadA, aminoglycoside 3′ ‐adenylytransferase gene; PpsbA, promoter and 5′ ‐UTR of psbA gene; CTB, coding sequence of non‐toxic cholera B subunit; VP1, poliovirus VP1 coding sequence; TpsbA, 3′ ‐UTR of psbA gene; trnI, isoleucyl‐tRNA; trnA, alanyl‐tRNA. PCR analysis of the spectinomycin‐resistant shoots expressing (b) CTB‐VP1n (L2.1, L2.6, L2.7) and (c) CTB‐VP1co (LS1, LS8, LS9, LCP2,LCP4) independent lines evaluated using 16sF/3M (denoted as d) and 23sF/3′ UTR (denoted as e) primer pairs. M: 1 kb DNA ladder, UT: untransformed wild‐type lettuce plants. Southern blot analysis of the PCR‐positive shoots expressing (d) DNA isolated from CTB‐VP1n (L2.1, L2.6, L2.7) and (e) CTB‐VP1co (LS1, LS8, LS9, LCP2 and LCP4) independent and untransformed plants was digested with HindIII followed by hybridization with trnI‐trnA flanking sequence probe (denoted as f) to develop 9.1 kb fragment for UT and 12.2 kb fragment from transplastomic lines.

Rare codons of poliovirus capsid protein VP1 (VP1) gene were replaced with highly preferred codons based on the hierarchy of codon usage of the most highly expressed chloroplast psbA genes (Kwon *et al*., 2016). The codon‐optimized sequence of VP1 was synthesized by GenScript, Piscataway, NJ. Subsequently, the native (*VP1n*) and codon‐optimized VP1 (*VP1co*) gene 906 bp fused with CTB (312 bp) and *VP1co* were sub‐cloned into the lettuce chloroplast transformation vector pLsLF (Figure 1a), using unique restriction enzyme sites *Nde*I and *PshA*I. Native sequence of the cholera non‐toxic subunit B (CTB), a transmucosal carrier protein, was used due to its high‐level expression in plant chloroplasts (Boyhan and Daniell, 2011; Ruhlman *et al*., 2010). *CTB‐VP1n, CTB‐VP1co* and *VP1co* genes are driven by the light‐regulated *psb*A promoter and 5′ UTR, and the transcripts are stabilized by the *psbA* 3′ UTR from the lettuce chloroplast genome. For selection of transplastomic shoots, the adenylyltransferase gene (*aadA*), which confers spectinomycin resistance, driven by the lettuce chloroplast Prrn promoter was inserted. To facilitate site‐specific integration of the expression cassette into the lettuce chloroplast genome, 16S rRNA‐trnI and trnA‐23S rRNA flanking sequences from the lettuce chloroplast genome were placed on either ends of the transgene cassettes (Figure 1a). The constructs pLsLF::CTB‐VP1n, pLsLF::CTB‐VP1co and pLsLF::VP1co (Figure 1a) were used to generate *CTB‐VP1n,CTB‐VP1co* and *VP1co* transplastomic lines respectively, using biolistic particle delivery system.

Independent transplastomic lines, CTB‐VP1n (*n* = 3) and CTB‐VP1co (*n* = 5) were selected on spectinomycin and analysed by PCR using two sets of primers including 16sF/3M and 3′ UTR/23sF (Figure 1a). The PCR product of all the independent lettuce lines expressing CTB‐VP1n (Figure 1b) and CTB‐VP1co (Figure 1c) showed site‐specific integration. Primers 16sF and 23sF anneal to the native chloroplast genome sequence upstream of the 16S trnI and downstream of the 23S trnA flanking sequences, thereby excluding the probability of off‐target integration of the expression cassettes into nuclear or mitochondrial genomes. The 16sF/3M and 3′ UTR/23sF primers generated 2.7 kb and 2.4 kb PCR products, from transplastomic lines, while no PCR product was observed in wild‐type untransformed lettuce plants (UT; Figure 1b,c). Once homology‐based integration was confirmed, the transplastomic lettuce plants were evaluated for homoplasmy or heteroplasmy employing Southern blot hybridization. The gDNA from CTB‐VP1n (L2.1, L2.6 and L2.7) and CTB‐VP1co (LS1, LS8, LS9, LCP2 and LCP4) transplastomic lines, and UT was digested with the restriction enzyme *Hind*III and probed with DIG‐labelled 1.12 kb trnI/trnA flanking region fragment. The selection of this enzyme is based on the fact that it digests the genomic DNA with reasonable frequency, but acts as a single cutter for the transgene cassette (not inside the CTB‐VP1n and CTB‐VP1co gene). In case of CTB‐VP1n (Figure 1D) and CTB‐VP1co (Figure 1e), the probe (Figure 1a) specifically detected the transformed chloroplast DNA fragment of 12.2 kbp size with no wild‐type hybridizing fragment at 9.1 kbp. Absence of 9.1 kb hybridizing fragment in transplastomic lines confirmed that the homoplasmy is achieved.

### Quantitation and pentamer assembly of CTB‐VP1

**Figure 2:**
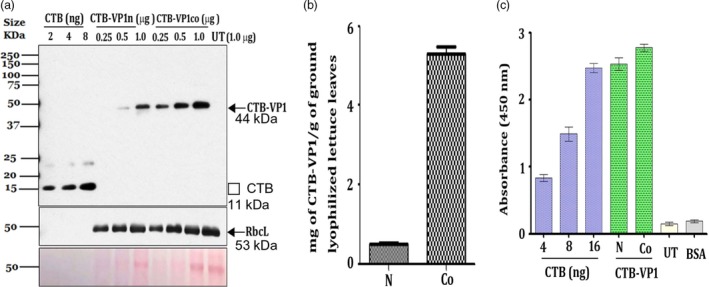
Immunoblot analysis and pentamer assembly of CTB in transplastomic lettuce leaves expressing native and codon‐optimized CTB‐VP1 (a) Western blot analysis for the comparison of the level of CTB‐VP1 in native (n) and codon‐optimized (co) lyophilized and ground lettuce leaves. Different concentrations of CTB (2, 4 and 8 ng) were loaded as standards. Ribulose bisphosphate carboxylase large chain (RbcL) and Ponceau S was used as loading control. (b) Densitometric analysis of the levels of native (n) and codon‐optimized (co) CTB‐VP1 in lyophilized and ground lettuce leaves (10 mg). Data are presented as mean ± SD of three independent experiments. (c) GM1‐binding assay of CTB‐VP1 and untransformed lettuce lines. Different concentrations (2, 1 and 0.5 μg/μL) of total soluble proteins of transplastomic and wild‐type untransformed lettuce were used. n: native, co: codon‐optimized, UT: untransformed wild‐type control; bovine serum albumin (BSA, 1%, w/v), CTB (4, 8 and 16 ng). BSA was used as negative control. Data are presented as mean ± SD of three independent experiments.

The homoplasmic transplastomic lines expressing CTB‐VP1n and CTB‐VP1co were transferred to the Daniell laboratory greenhouse at the University of Pennsylvania. Leaves were harvested from fully grown lettuce, lyophilized and ground as described previously (Kwon and Daniell, 2016). The protein accumulation and integrity of CTB‐VP1n and CTB‐VP1co in lyophilized and ground lettuce leaves was analysed by western blots (Figure 2a). Western blots showed the CTB‐VP1 44 kDa polypeptide (Figure 2a), representing the monomer; with equal loading using endogenous Rubisco chloroplast protein and Ponceau S, native VP1 protein was barely visible but codon‐optimized CTB‐VP1 gene showed very high level of expression. Intensities of the native or codon‐optimized CTB‐VP1 gene expression were compared with known amounts of CTB standard. The western blot analysis clearly indicates that the expression of codon‐optimized VP1 (CTB‐VP1co) was 9.29–15.36‐fold higher than CTB‐VP1n (Figure 2B). The data presented in mean of three independent experiments. GM1‐binding ELISA was performed to evaluate the formation of pentameric structures of the CTB‐VP1n and CTB‐VP1co protein expressed in chloroplasts. As shown in Figure 2C, both native and codon‐optimized CTB‐fused VP1 protein showed comparable absorbance to CTB (positive control), indicating that CTB‐VP1 fusion protein has a pentameric assembly and can bind to GM1 as strongly as purified CTB protein and thus fulfil the requirement for oral antigen delivery. Observation of pentameric assembly of CTB‐VP1 fusion protein as efficient as control CTB suggests that fusion protein three times larger than CTB did not interfere with assembly of pentamers required for GM1 binding.

### VP1 assembly as Virus‐like particles

**Figure 3:**
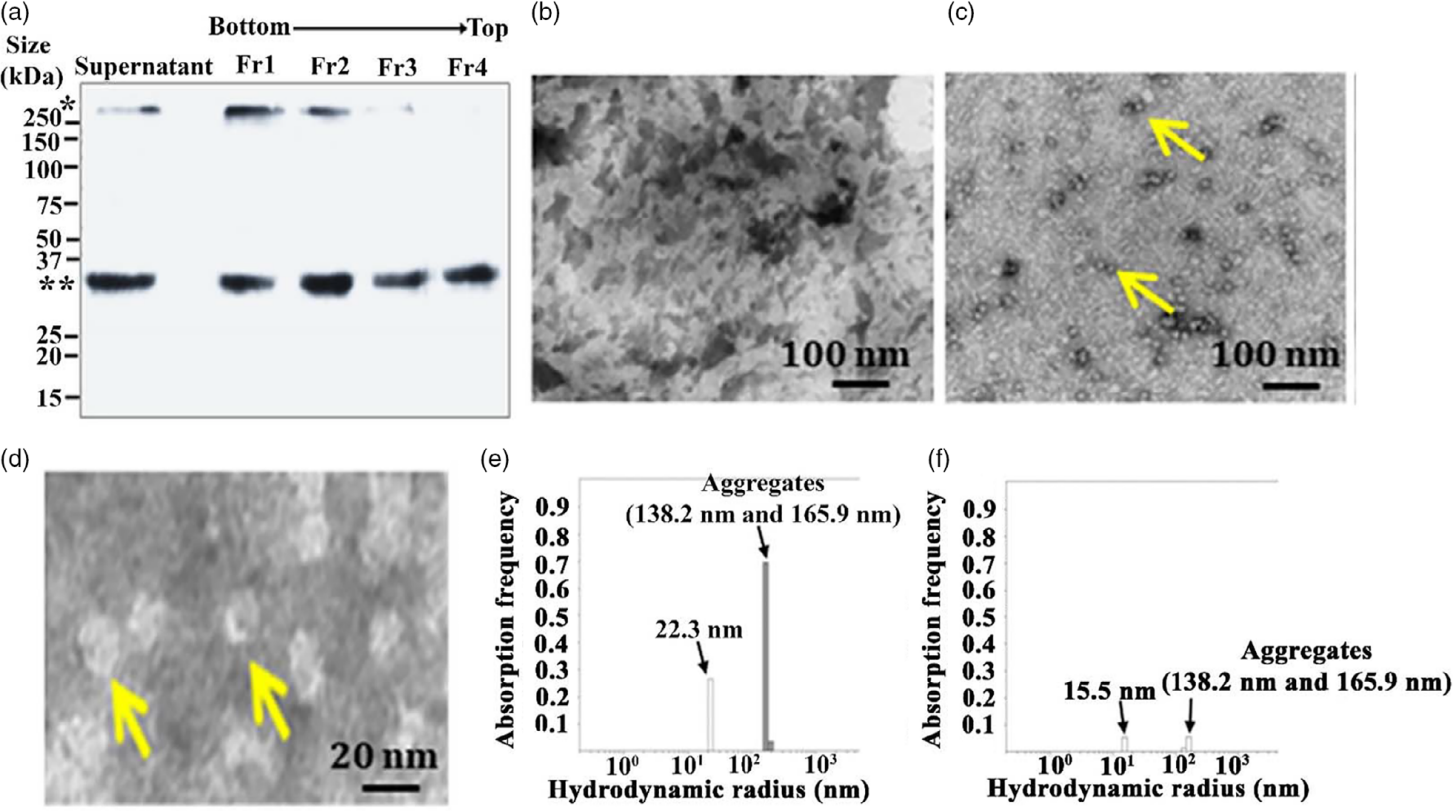
Assessment of polio VP1 VLPs in transplastomic lettuce leaves. (a) Western blot analysis of VP1 crude supernatant (S) and enriched fractions using anti‐VP1 antibody. Lettuce chloroplast extracts were sedimented through sucrose gradient (20%–40%) for fractionation. Four fractions were collected from bottom to top. The expected VP1 monomer size is 33 kDa (**). The putative VLPs are indicated as asterisk (*). Transmission electron microscopic analysis of fraction 1 (b) untransformed wild‐type 100 nm resolution, (c) VP1 transplastomic line 100 nm resolution and (d) VP1 transplastomic line 20 nm resolution, visualized by negative staining. Examples of some VLPs are highlighted with yellow solid arrows. Examination of polio VLP size distribution by DLS (e, f) shows histograms generated from the enriched fraction 1 of the VP1 expressing lines and the untransformed wild‐type, respectively.

To confirm assembly of the VP1 protein into higher order structures or VLPs, lettuce chloroplast extracts were sedimented through a sucrose gradient. Most of the VP1 protein concentrated in the fraction 1–4 (bottom to top). All the four fractions along with the crude supernatant (S) showed the expected size polypeptide (33 kDa) when immunoblotted with anti‐VP1 antibody (Figure 3a). Further TEM examination of fraction 1 confirmed the presence of large amount of assembled VLPs in VP1 chloroplasts (Figure 3e,f), but not in extracts of chloroplasts from untransformed leaves (Figure 3d). Magnified image of the regular spherical‐shaped VP1 VLP is shown in Figure 3c; 20 nm size bar shows the VLP size to be around 20 nm. Dynamic Laser Scan (DLS) analysis of the VP1 transplastomic lines (Figure 3b) confirmed the VP1 VLP size to be 22.3 nm for the monomer and 138.2 and 165.9 for multimeric VLPs. In contrast, untransformed plants did not show hydrodynamic radius of 22.3 nm, while that for 138.2 and 165.9 was below 0.05 (Figure 3c), thus considered as background noise due to interference of solvent or other proteins.

### Antimicrobial peptides induced higher VP1‐specific antibody titres

**Figure 4:**
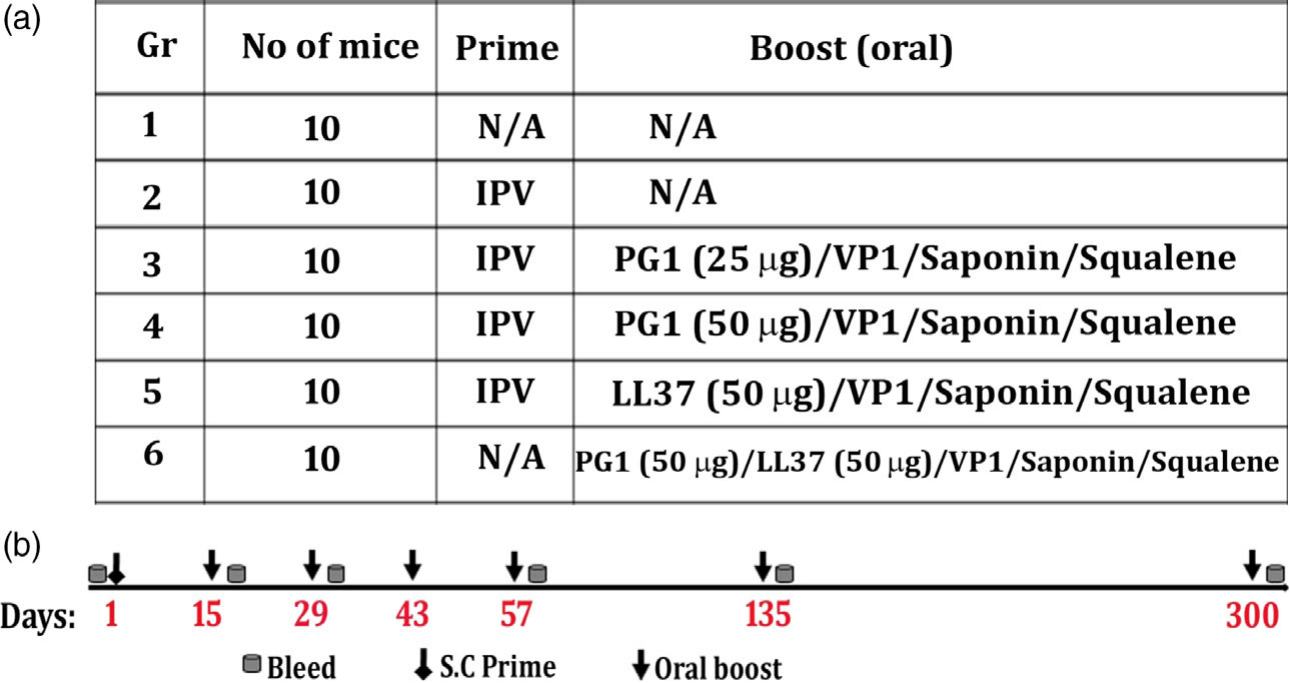
Design and grouping of *in vivo* polio vaccine study. (a) Female CD1 mice were randomly divided into six groups (*n* = 10). Lyophilized and ground lettuce cells expressing 1 μg or 25 μg of CTB‐VP1co, plant‐derived adjuvants (Saponin and/or Squalene) and antimicrobial peptides (LL37 and PG1) were used in this study. (b) All groups of mice except group 1 and 6 were subcutaneously primed with IPV at day 0. Group 3–6 were orally boosted with lyophilized and ground lettuce cells expressing CTB‐VP1co once every 2 week for 2 months.

**Figure 5:**
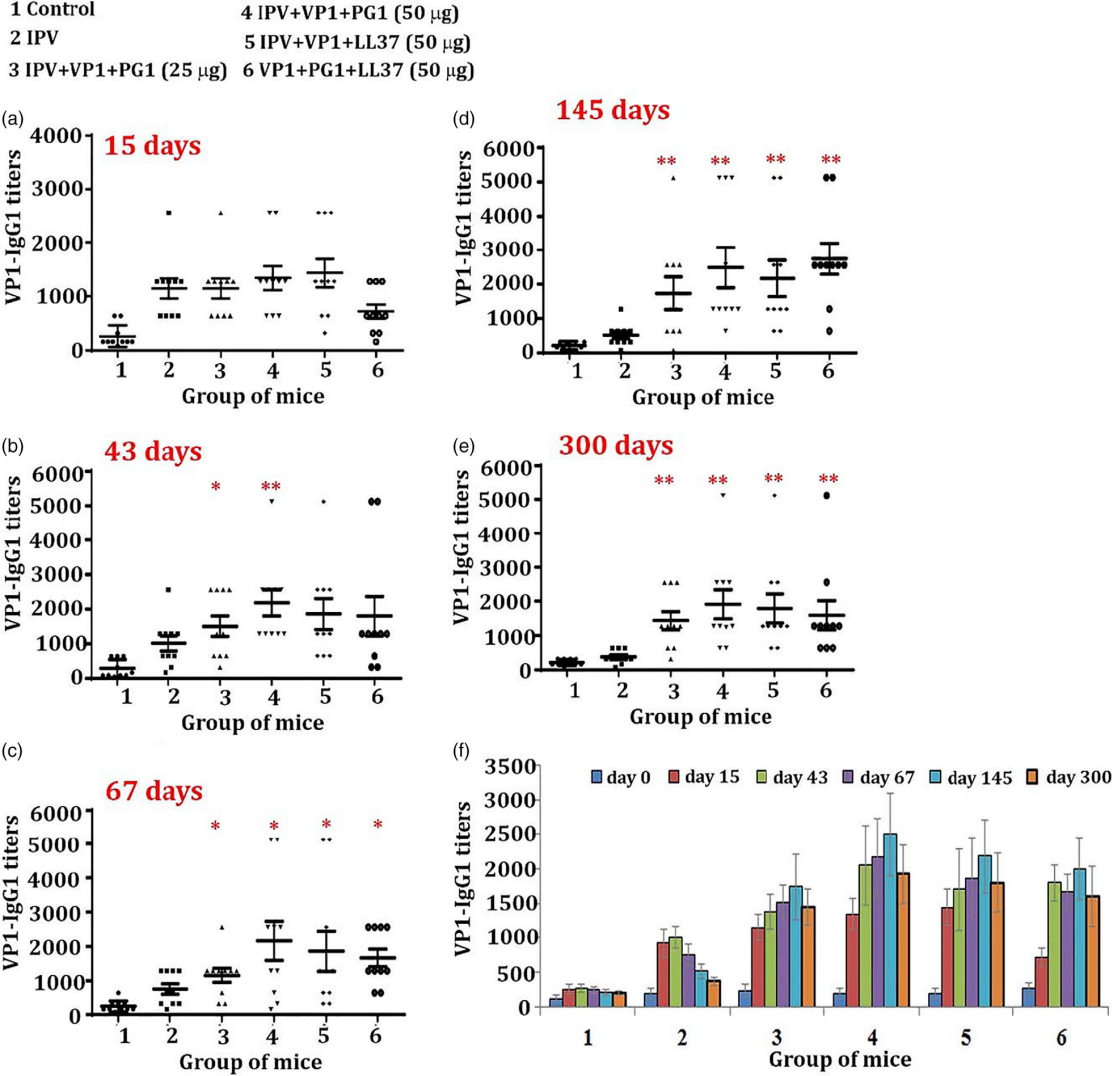
Evaluation of serum VP1‐IgG1 antibody titres after oral or subcutaneous polio vaccination in female CD1 mice. Immunological response of mice (*n* = 10/group) vaccinated with single dose of inactivated poliovirus vaccine (IPV) or boosted lettuce CTB‐VP1co and adjuvants are shown. (a–e) VP1‐IgG1antibody titres at different time points: (a, b) weekly boosts and sera samples collected on days 15 and 43; (c, d, e) monthly boosts and samples collected on days 67, 145 and 300. (f) Kinetic of serum VP1‐IgG1 antibody titres upon oral and/or subcutaneous polio vaccination. Serum VP1‐IgG1 antibody titres were assayed for a period of 300 days by direct ELISA in 96 well plates pre‐coated with purified VP1 protein (10 μg/mL). Group 1: untreated, Group 2: single dose of IPV, Group 3–6: IPV prime, boosted with codon‐optimized CTB‐VP1 (25 μg) *in plant* with adjuvants (saponin/squalene) and antimicrobial peptides (detailed in Figure 4). Results are shown as individual reciprocal endpoint antibody titres and mean ± SEM. One‐way ANOVA showed significant differences between groups (*P* < 0.0001) and *post hoc* comparisons by *t‐test* showed significant differences between specific treatment groups and control group (Group 2). (*P < 0.05,**P < 0.01).

**Figure 6:**
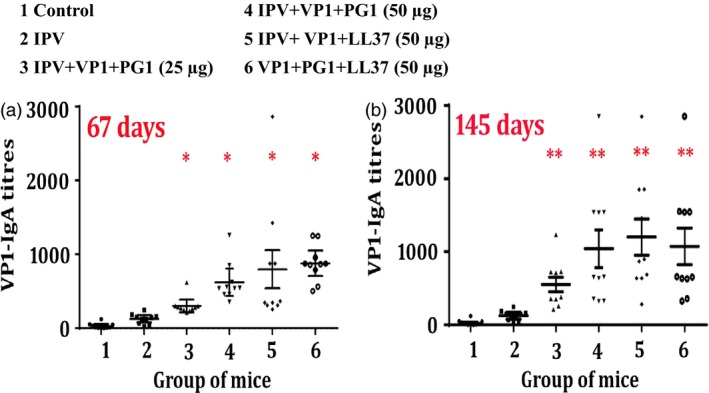
Evaluation of serum VP1‐IgA antibody titres after oral or subcutaneous polio vaccination in female CD1 mice. Immunological response of mice (*n* = 10/group) vaccinated with single dose of inactivated poliovirus vaccine (IPV) or boosted lettuce CTB‐VP1co and adjuvants are shown. VP1‐IgA antibody titres of sera sample collected on (a) 67 and (b) 145 day respectively. Serum VP1‐IgA antibody titres were assayed by direct ELISA in 96 well plates pre‐coated with purified VP1 protein (10 μg/mL). Group 1: untreated, Group 2: single dose of IPV, Group 3–6: IPV prime, boosted with CTB‐VP1co (25 μg) *in plant* with adjuvants (saponin/squalene) and antimicrobial peptides (detailed in Figure 4). Results are shown as individual reciprocal endpoint antibody titres and mean ± SEM. One‐way ANOVA showed significant differences between groups (*P* < 0.0001) and *post hoc* comparisons by *t*‐test showed significant differences between specific treatment groups and control group (Group 2). (*P < 0.05,**P < 0.01).

In order to develop new vaccine formulations and reduce number of CTB‐VP1 oral boosters, we used plant adjuvants saponin and squalene and immune‐modulating antimicrobial peptides protegin‐1 and LL37. All adjuvants tested have been previously approved for clinical evaluation. In addition, we wanted to understand if oral adjuvants along with CTB‐VP1 antigen would have any additive effect to enhance oral priming or boosting. Mice boosted with CTB‐VP1co adjuvanted with saponin, squalene and either 25 μg PG‐1 (group 3), 50 μg PG‐1 (group 4), 50 μg LL37 (group 5), or 50 μg LL37 and 50 μg PG‐1 (Figure 4) produced significantly higher VP1‐IgG1 (1504, 2176, 1696, 1536) at day 43 after priming than a single dose of IPV (1008, Figure 5). VP1‐IgA titres were significantly higher (200, 340, 640, 704) in mice boosted with lyophilized plant cell expressing CTB‐VP1co at day 67 than a single dose of IPV (88, Figure 6). Furthermore, these levels stayed high over the period of 300 days in mice boosted with plant cells expressing CTB‐VP1co, suggesting three plant boosters are highly effective and the multiple plant cell boosters reported previously (Chan *et al*., 2016; Xiao and Daniell, 2017) have minimal role in attaining and maintaining immunogenicity and neutralize different poliovirus serotypes. In contrast, a single IPV dose resulted in gradual decline in VP1‐IgG1 (from 1008 to 520; Figure 5) and negligible VP1‐IgA antibody levels (Figure 6). Kinetic analysis of VP1‐IgG1 antibody titres confirmed that titres increased from 15th day and remained high throughout the course of study (Figure 5F) in CTB‐VP1co boosted mice, but the counterparts in IPV single dose group remained negligible, confirming that human antimicrobial peptides (PG‐1, LL37) are highly effective as adjuvants and have additive effects.

### Antimicrobial peptide induced higher poliovirus‐neutralizing titres

**Figure 7:**
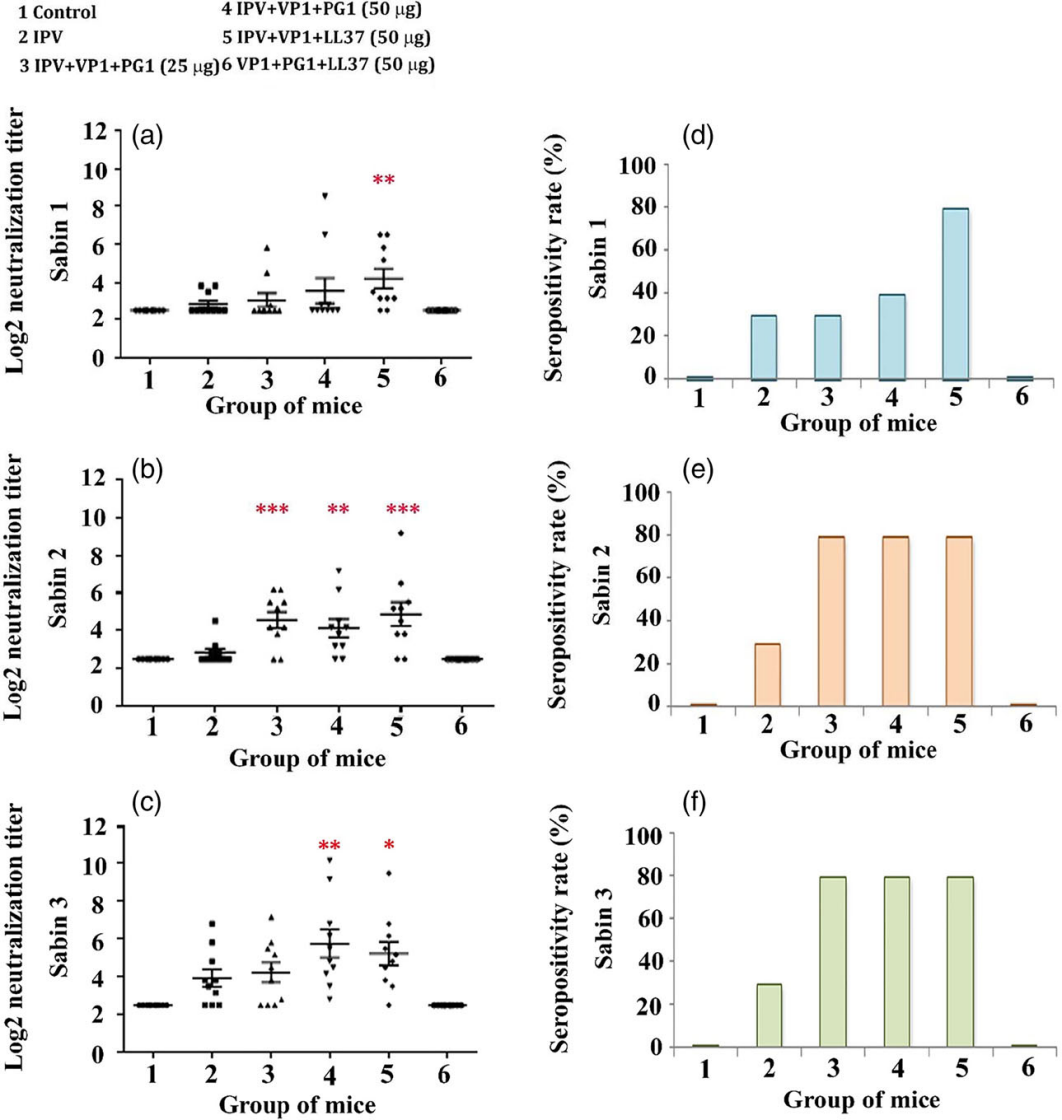
Determination of poliovirus‐neutralizing titres and seropositivity rate of Sabin 1‐, 2‐ and 3‐neutralizing titres following polio vaccination. Virus‐neutralizing antibody titres of female CD1 mice sera (*n* = 10/group) collected on 67th day following IPV priming (Group 2) and/or boosting CTB‐VP1co (25 μg) *in planta* with adjuvants (saponin/squalene) and antimicrobial peptides (Group 3–6). Results are shown as mean neutralizing titre ± SEM of individual mice against all three Sabin strains (a) Sabin 1 (b) Sabin 2 and (c) Sabin 3.The serum dilution of reciprocal titre >log_2_ (titre) of 2.5 was considered positive. One‐way ANOVA showed significant differences between groups (*P* < 0.0001) and *post hoc* comparisons by *t‐test* showed significant differences between specific treatment groups and control group (Group 2). (*P < 0.05, **P < 0.01, ***P < 0.001). The seropositivity rate of poliovirus‐neutralizing antibodies was determined by the number of mice with seroprevalence (neutralizing antibody log2 (titre ≥3) in comparison to the total number of mice in each group boosted with codon‐optimized CTB‐VP1 or IPV single dose (group 2). The seropositivity rate of neutralizing titres against Sabin strains 1 (d), strains 2 (e) and strains 3 (f) is shown.

To determine the efficacy of the new vaccine formulation comprising of the antimicrobial peptides, as adjuvants in neutralizing antibody titres, blood samples (collected post 4th oral boosting) from all experimental groups were tested in a double‐blind manner and in triplicate at CDC. As shown in Figure 7, the vaccine formulation composed of saponin, squalene and 50 μg LL37 produced highest neutralizing antibody titres and seropositivity rate against Sabin 1, but similar neutralizing antibody titres and seropositivity rates against Sabin 2 and Sabin 3 were observed in group of mice orally boosted with formulations containing 25 μg (group 3) or 50 μg PG‐1 (group 4) or 50 μg LL37 (group 5). Mice orally boosted with CTB‐VP1co plus four adjuvants without priming elicited significantly higher VP1‐IgG1 and VP1‐IgA antibody titres but no protective neutralizing antibody titres. These findings demonstrated that without IPV priming, only oral boosting with plant‐made vaccine antigen, irrespective of any formulation used, is not adequate to induce any neutralizing antibodies. In case of Sabin 1 and Sabin 2, a single dose of IPV resulted in poor seropositivity (<20% and <40%, respectively), but showed ~70% seropositivity against Sabin 3. LL37 had higher seropositivity than PG‐1 against Sabin 1, but both were equally protective against Sabin 2 and Sabin 3 (80%–100% protection; Figure 7).

## Discussion

Continued use of OPV resulted in vaccine‐associated paralytic poliomyelitis (VAPP) and the emergence of genetically divergent, neuro‐virulent vaccine‐derived polioviruses (VDPVs). Several cases of VDPVs including type 1 and 2 have been reported in more than 15 countries (Diop *et al*., 2015; Jorba *et al*., 2017), but large majority of VDPV case‐isolates were type 2. In order to address these concerns, trivalent OPV (tOPV) is replaced with bivalent, excluding serotype 2, preceded by introduction of at least one dose of injectable IPV (Previsani *et al*., 2017). So, the key question is the adequacy of single dose IPV to confer protection against wild polio virus type 2 (WPV2). Unfortunately, recent publications raise important concerns. IgG1 titres in single or double dose IPV decreased dramatically in one month and reached to background levels after 400 days (Xiao and Daniell, 2017), with minimal mucosal immunity (Chan *et al*., 2016; Xiao and Daniell, 2017), suggesting that IPV would have minimal impact on containment of cVDPV or prevent silent polio outbreaks. In countries with open sewer system, reinfection with poliovirus is a major challenge and lack of gastrointestinal mucosal immunity with IPV would pose additional challenges. In 2002, the European Region was certified polio‐free and therefore polio vaccine doses were reduced resulting in favourable conditions for VDPV1 emergence and circulation, leading to the outbreak in Ukraine (Khetsuriani *et al*., 2017). Therefore, in this study we report development of an affordable virus and cold chain‐free booster vaccine for long‐term maintenance of gastrointestinal immunity and protection against all three poliovirus serotypes.

Orally delivered protein drugs are digested by acids and enzymes in the stomach, and proteins that escape digestion are blocked from absorption in the gut by impermeable gut epithelium. Both these challenges are addressed when protein drugs are expressed at high levels in chloroplasts and bioencapsulated within plant cells that protect them from digestion in the stomach. However, commensal bacterial enzymes digest plant cell wall and release protein drugs in the gut lumen. When CTB is fused to vaccine antigens, they cross the gut epithelium and are delivered to the immune system. In addition to conferring gastrointestinal immunity, plant cell‐based drug delivery is highly affordable as it eliminates prohibitively expensive fermentation and purification processes that are used in current vaccines.

In this study, site‐specific integration of *CTB‐VP1* gene was confirmed by PCR products generated from unique primer sets annealing to the native chloroplast genome and the transgene cassette. Homoplasy was determined by absence of wild‐type untransformed genomes (9.12 kbp hybridizing fragment) in Southern blots. Quantitation of CTB‐VP1 in lyophilized lettuce cells using CTB standard showed 9.29–15.36‐fold higher expression in codon‐optimized gene than the native viral gene. The enhanced expression level of CTB‐VP1 in this study through codon optimization and expression in lettuce chloroplasts could advance virus‐free and cold chain‐free oral polio booster vaccine to the clinic. Pentameric assembly of CTB‐VP1 fusion protein expressed in lettuce chloroplasts was evident from GM1 binding ELISA assay, which is an important requirement for oral vaccine antigen delivery through gut epithelium.

Since vaccine antigens can be stored in lyophilized plant cells at ambient temperature for several years without losing their efficacy (Herzog *et al*., 2017; Su *et al*., 2015), a further reduction in cost is achieved by elimination of cold storage and transportation. Clinical grade materials can be produced in a cGMP facility at Fraunhofer (Herzog *et al*., 2017). In order to file Investigational New Drug application with FDA, pharmacokinetics, pharmacodynamics, safety, toxicology and tolerability studies of orally delivered protein drugs bioencapsulated in plant cells have been conducted (Daniell lab, unpublished). These advances should facilitate regulatory approval, clinical studies and affordable vaccine to eradicate polio.

Assembly of VP1 into higher order structures (VLPs) was examined by TEM of negatively stained fraction 1, after verification with VP1 antibody. We observed particles of 22.3 nm VP1 monomers and 138.2 and 165.9 nm multimers in transplastomic lines expressing VP1 but not in untransformed lines. Multimer VLPs of VP1 were observed in greater frequency than monomer VLPs. VLP formation in chloroplasts is not a surprising observation because we reported prM/E dengue VLPs expressed in lettuce chloroplasts (Kanagaraj *et al*., 2011). Likewise, when L1 gene was expressed in chloroplasts, it self‐assembled into VLPs suggesting no requirement of endoplasmic reticulum processing for VLPs formation (Fernandez‐San Millan *et al*., 2003; Lenzi *et al*., 2008). However, for immunization studies CTB‐VP1 was used because VP1 antigen without fused transmucosal carrier would not be efficiently delivered to the immune system. When injected, VLPs could enhance uptake by dendritic cells (DC) via macropinocytosis and endocytosis that play a central role in activating innate and adaptive immune responses. However, upon oral delivery, they should cross the gut epithelium first.

Although the “edible vaccine” concept was pioneered with the anticipation of delivering vaccine antigens orally, key immunological questions remain unaddressed. Do orally delivered antigens induce immunity by producing antibodies or do they suppress immunity by blocking antibody formation and inducing tolerance? A large number of recent publications on antigens fused with CTB have shown tolerance induction and immune suppression in the absence of injectable priming with antigens (Herzog *et al*., 2017; Kwon *et al*., 2018; Ruhlman *et al*., 2010; Sherman *et al*., 2014; Su *et al*., 2015; Verma *et al*., 2010). Therefore, in this study, we explored various plant adjuvants (Saponin/Squalene) or antimicrobial peptides (PG1 and LL37) to enhance oral priming, along with lyophilized plant cells expressing CTB‐VP1, with or without IPV injectable priming.

Mice boosted with CTB‐VP1co adjuvanted with saponin, squalene and either 25 μg PG‐1 (group 3), 50 μg PG‐1 (group 4), 50 μg LL37 (group 5) or 50 μg LL37 and 50 μg PG‐1 produced significantly higher VP1‐IgG1 (1504, 2176, 1696, 1536) on day 43 than a single dose of IPV (1008). On day 300, single dose IPV group VP1 titres reached almost the background levels whereas all groups boosted with CTB‐VP1co plant cells steadily maintained VP1 IgG1 titres. VP1‐IgA titres were significantly higher (200, 340, 640, 704) in mice boosted with lyophilized plant cells expressing CTB‐VP1co on day 67 than a single dose of IPV with minimal IgA titre. VP1 IgA titres continued to increase during the entire study period in mice groups boosted with plant cells expressing CTB‐VP1. So, these studies confirm that three oral boosters are more than adequate to maintain VP1 IgG1 and IgA‐specific titres and fifteen boosters used in previous study (Chan *et al*., 2016; Xiao and Daniell, 2017) were unnecessary and have minimal role in attaining and maintaining immunogenicity and protection against poliovirus serotypes. In addition, human antimicrobial peptides (PG‐1, LL37) are highly effective as adjuvants and have additive effects in boosting immunity. All adjuvants used in this study have been approved for clinical applications. Lack of IgA (mucosal immunity) and decline in IgG1 VP1‐specific titres after 1 month calls into question WHO recommendation of a single dose of IPV after withdrawal of OPV2. In contrast, three oral boosters are adequate to achieve high‐level IgG1/IgA titres and longer lasting mucosal and systemic immune responses.

All serum samples from the Daniell laboratory were tested in a double‐blind manner and in triplicate at the Center for Disease Control, Atlanta, USA. Vaccine formulation composed of all adjuvants produced the highest neutralizing antibody titres and seropositivity rate against Sabin 1, clearly demonstrating additive effect of both immune‐modulating antimicrobial peptides. Each antimicrobial peptide or different doses showed similar seropositivity against Sabin 2 and Sabin 3 serotypes. Interestingly, the most problematic Sabin 2, recently withdrawn by WHO showed the highest Log2 neutralization titres, meeting the most urgent global need. In sharp contrast, IPV single dose generated the lowest neutralization titres and seropositivity against all three serotypes. Equally striking observation is the lack of neutralization titre or seropositivity for mice group not primed with IPV, although this group showed the highest VP1‐specific IgA and IgG1 titres. This is an important lesson for vaccine development studies that both immune responses and seropositivity rates or pathogen/toxin challenge data are compared before making definitive conclusions on vaccine efficacy. These observations underscore the reality that injectable priming is an important requirement and none of the oral adjuvants we tested were capable of priming. In case of polio vaccine, WHO has recommended a single IPV vaccination and therefore only boosters are required. Plant cell‐made vaccine antigens are ideal for disease where global population is already primed with single dose of vaccines or natural exposure to pathogens (polio is detected in open sewer systems). In the absence of such priming, plant cells are not suitable for vaccination against infectious diseases. Gut is a tolerogenic environment and the key role of the gut is to develop tolerance to presented antigens. Therefore, gut is ideal to deliver protein drugs to circulation and block antibody formation.

## Materials and Methods

### Creation of CTB‐VP1transplastomic lettuce lines

Poliovirus capsid protein *VP1* derived from Sabin 1 coding sequences (Genbank accession number AY184219; 906 bp) was codon‐optimized (*VP1co*) using the in‐house tool (Kwon *et al*., 2016) and synthesized by GenScript, Piscataway, NJ. The *VP1co* and native *VP1n* fused with cholera toxin B subunit (*CTB*; 312 bp) and *VP1co* genes were cloned into the lettuce chloroplast transformation vector pLsLF. Lettuce leaves which were 3 to 4 weeks old were bombarded with gold particles (0.6 μm) coated with the respective plasmid DNA (pLsLF::*CTB‐VP1n,* pLsLF::*CTB‐VP1co* and pLsLF:: *VP1co*) using the biolistic device PDS1000/He following the previously described method (Kumar and Daniell, 2004; Ruhlman *et al*., 2010). Subsequent regeneration of transplastomic lettuce plants were done in spectinomycin selection media following the method described previously (Ruhlman *et al*., 2010).

### Transgene cassette integration analysis

Shoots (*CTB‐VP1n* and *CTB‐VP1co*) developed after the second round of spectinomycin selection were analysed for transgene integration by PCR. Genomic DNA (gDNA) from lettuce shoots expressing *CTB‐VP1n* and *CTB‐VP1co* and wild‐type was extracted following previously reported protocol (Allen *et al*., 2006). Site‐specific integration assay for *CTB‐VP1n* and *CTB‐VP1co* genes was performed by PCR amplification of gDNA obtained from each line individually using 16sF (5′CAGCAGCCGCGGTAATACAGAGGATGCAAGC3′)/3M (5′CCGCGTTGTTTCATCAAGCCTTACGGTCACC3′) and 3′ UTR (5′AGGAGCAATAACGCCCTCTTGATAAAAC3′)/23sF (5′TGCACCCCTACCTCCTTTATCACTGAGC3′) primer sets (Figure 1a). Primer 16sF anneals to the chloroplast genome upstream of the 16S‐trnI gene outside the transgene cassette integration site and primer 3M anneals to the aadA gene. The 3′ UTR primer anneals to the 3′ UTR region of the TpsbA region and 23sF to the chloroplast genome upstream of the 23S‐trnA gene outside the vector integration site. PCR conditions were initial denaturation at 95°C for 5 min, annealing at 56°C for 30 s, extension at 72°C for 30 s (34 cycles), followed by final extension step at 72°C for 5 min. The PCR products were electrophoresed in 1.0% agarose gels and stained with ethidium bromide.

Southern hybridization was carried out according to the previously published protocol of Su *et al*. (2015), to determine homo/heteroplasmy of lettuce transplastomic lines. Briefly, 5 μg of gDNA isolated from transplastomic and wild‐type lettuce plants was digested overnight with *Hind*III HF (NEB) at 37°C, electrophoresed on 0.7% TAE‐agarose and transferred to a nylon membrane (Hybond‐N+, Amersham). Blotted DNA fragments were hybridized with DIG‐labelled probe (1.12 kb *trnI*/*trnA* flanking region) at 42°C for 16 h, subsequently the membrane was washed and developed as per the previously described protocol (Kanagaraj *et al*., 2011). The Southern blot confirmed lettuce plants were transferred to greenhouse.

### Quantitation of CTB‐VP1

Total plant protein from the greenhouse grown young leaves or the powdered lyophilized plant cells of transplastomic lettuce expressing *CTB‐VP1n* and *CTB‐VP1co,* and wild‐type were extracted in the protein extraction buffer (100 mm NaCl, 10 mm EDTA, 200 mm Tris‐Cl pH 8.0, 0.05% (v/v) Tween‐20, 0.1% SDS, 14 mm β‐ME, 400 mm sucrose, 2 mm PMSF and proteinase inhibitor cocktail). Total protein extracts were analysed by 10% SDS‐PAGE. Western blot analysis was performed by rabbit anti‐CTB polyclonal primary antibody (GenWay Biotech Inc, San Diego, CA) in a dilution of 1:10 000 followed by detection with a goat anti‐rabbit IgG‐HRP secondary antibody (1: 4000 dilution; SouthernBiotech, Birmingham, AL) and developed using the ECL chemiluminescence (GE healthcare, Stevenage, UK). CTB (Cat# GWB‐7B96E4; GenWay Biotech Inc, San Diego, CA) was used as positive control and served as standard for quantitative analysis of the blot with Image J software (IJ 1.46r; NIH). For loading controls, protein‐blotted membrane was stained with Ponceau S (Sigma, P‐3504) prior to immunoprobing with anti‐CTB antibody and anti‐RbcL antibody (Cat# AS03 037, Agrisera, 1:5000) was used.

### GM1‐ganglioside receptor‐binding assay

CTB–GM1‐binding assay was performed to test the efficacy of lettuce chloroplast‐derived CTB‐VP1n and CTB‐VP1co fusion protein to form pentamers and corresponding binding to the GM1‐ganglioside receptor. The experiment was performed as described previously (Ruhlman *et al*., 2007; Worstell *et al*., 2016). The 96‐well microtitre plates were coated with monosialo‐gangliolside‐GM1(Sigma G‐7641; St. Louis, MO) by incubating the plate with 100 μL of GM1 (3.0 μg/mL) in the coating buffer (15 mm Na_2_CO_3_, 35 mm NaHCO_3_ pH8.6) at 4°C overnight. Plates were washed thrice with phosphate buffered saline with Tween‐20 (PBST) and wells were blocked with 3% non‐fat dry milk in PBST for 2 h at 37°C. Plates were incubated overnight at 4°C with different concentrations (2 μg/μL, 1 μg/μL and 0.5 μg/μL) of total soluble protein from transplastomic lettuce expressing *CTB‐VP1n* and *CTB‐VP1co,* or wild‐type. Different concentrations of CTB (4, 8 and 16 ng) were used as positive control and BSA (1%) was used as the negative control. Subsequently, the interaction was detected by rabbit anti‐CTB polyclonal antibody (GeneWay Biotech Inc, San Diego, CA) in a dilution of 1:10 000 followed by 1: 4000 goat anti‐rabbit IgG‐HRP secondary antibody (SouthernBiotech, Birmingham, AL), and visualized at 450 nm by adding 3,3′,5,5′‐tetramethyl benzidine substrate.

### Enrichment of plant VLPs and TEM evaluation

Enrichment of plant VLPs and TEM examination of the VP1co and wild‐type was carried out using the previously described protocol (Kanagaraj *et al*., 2011). Briefly, lettuce chloroplast was isolated by homogenizing the deveined mature leaves (10 g) in a Waring blender containing 50 mL ice cold buffer (25 mm HEPES–KOH, pH 7.7, 330 mm sorbitol, 2 mm EDTA, 1 mm MgCl_2_, 1 mm MnCl_2_, 0.25% BSA and 0.1% ascorbic acid). Homogenate was centrifuged at 1,000 × g for 5 min and filtered through muslin cloth. Isolated chloroplast was washed twice with wash buffer (PBS, pH 7.5, 0.5 m NaCl, 2 mm EDTA and protease inhibitor) and homogenized. Further, these samples were sonicated and centrifuged 10,000 × g for 20 min at 4°C for chloroplast protein extraction. Supernatant was filtered through 0.2 μm PES syringe filter and centrifuged at 15 000 ***g*** for 3 h at 10°C by layered onto a 20%–40% continuous sucrose gradient (Waheed *et al*., 2011). Four fractions (1, 2, 3 and 4, bottom to top) along with the crude supernatant (S) were dialysed to remove sucrose and immunoblotted using 1:1000 rabbit anti‐VP1 polyclonal antibody (Alpha Diagnostic Intl. Inc., SanAntonio, TX). Negative staining was performed by incubating 20 μL of the fraction 1 (10 mg/mL, diluted with wash buffer) with 2% uranyl acetate (w/v) solution for 1 min on 400 mesh (Electron Microscopy Sciences, Hatfield, PA) carbon‐coated copper grids. Grids were imaged under a JEOL 1011 transmission electron microscope at 80 kV and images were captured using AMT Image Capture Engine software (Advanced Microscopy Techniques, Corp. Danvers, MA). Dynamic Laser scattering (DLS) was performed to determine the particle diameter.

### Mice and immunization study

Immunization studies were performed as per our previous publications (Chan *et al*., 2016; Xiao and Daniell, 2017). Briefly, 6‐week‐old CD‐1 female mice were purchased from Charles River Laboratories and were randomly divided into six groups (Figure 4A). Group 1 was untreated. All mice from group 2 to 5 were subcutaneously primed with IPV, and mice from group 3 to 5 were orally boosted 14 days post‐priming with 20 mg of lyophilized codon‐optimized lettuce plant cells expressing VP1 (CTB‐VP1co) formulated with saponin and squalene plus 25 μg PG‐1 (Protegrin‐1, ANASPEC, Fremont, CA) (group 3), 50 μg PG‐1 (group 4) or 50 μg LL37 (Human antimicrobial peptide, ANASPEC, Fremont, CA) (group 5). Mice in group 6 were orally boosted with lettuce plant‐made CTB‐VP1co protein (20 mg) with all four adjuvants but without IPV priming. Oral boosts were performed every 2 weeks for duration of 2 months. Three months after last boosting, mice in group 3–6 were boosted with VP1 formulated with different adjuvants. Blood was collected 1 day prior to priming and 10 days after each boost (Figure 4b).

### Determination of IgG1 and IgA responses by ELISA

All serum samples were heat inactivated at 56°C for 30 min to inhibit complement activity as previously described (Chan *et al*., 2016). Serum VP1‐specific IgG1 and IgA antibodies were assayed by direct ELISA following previously reported protocol (Davoodi‐Semiromi *et al*., 2010). ELISA plates (96‐well) were pre‐coated with 10 μg/mL purified VP1 protein and twofold dilutions of heat‐inactivated individual serum samples were added. The starting dilutions of serum samples were 1:400 for VP1‐IgG1, and 1:40 for VP1‐IgA. Plates were incubated at 4°C overnight, and probed with anti‐mouse secondary antibodies. The absorbance and antibody titres were determined as previously described (Chan *et al*., 2016; Frey *et al*., 1998). All serum samples were tested in triplicate and the results are shown as individual antibody titre ± SEM.

### Poliovirus Sabin 1, 2, 3 neutralization assay

As described previously, the neutralization assays were performed at Centers for Disease Control (CDC) using a modified micro‐neutralization assays for antibodies to Sabin strains type 1, 2 and 3. The individual serum samples from control and experimental groups were collected 10 days after 4th oral boosting with VP1 formulations and tested randomly and blindly for neutralization assays as previously described (Chan *et al*., 2016; Xiao and Daniell, 2017). The reciprocal titre at which no virus neutralization was detected (negative) was recorded as the log_2_ (titre) of 2.5 or negative (Figure 7a–c); a log_2_ titre of ≥3 was considered protective. Individual titres for each group are plotted, and the bar represents mean neutralizing titre ± SEM.

### Statistical analysis

Data are reported for individual mice and groups; mean of three independent experiments ± SEM is provided for each group. One‐way ANOVA and Student's *t*‐test (GraphPad Prism version 6) were performed to identify significant differences between groups. The *P*‐value of <0.05 was considered significant.

## Conflict of interest

Henry Daniell, has several patents in the field of chloroplast genetic engineering and production of biopharmaceuticals in chloroplasts. Google Scholar link is provided http://scholar.google.com/citations?user=7sow4jwAAAAJ&hl=en for full disclosure of these patents. However, he has no specific financial conflict of interest to declare.
